# Glutamatergic synaptic currents of nigral dopaminergic neurons follow a postnatal developmental sequence

**DOI:** 10.3389/fncel.2015.00210

**Published:** 2015-05-29

**Authors:** Edouard Pearlstein, Laurie-Anne Gouty-Colomer, François J. Michel, Robin Cloarec, Constance Hammond

**Affiliations:** ^1^UMR 901, Aix-Marseille UniversitéMarseille, France; ^2^Institut de Neurobiologie de la Méditerranée, Inserm UMR 901Marseille, France

**Keywords:** substantia nigra, dopaminergic neurons, development, AMPA receptors, NMDA receptors, EPSC, NR2D subunit, patch clamp

## Abstract

The spontaneous activity pattern of adult dopaminergic (DA) neurons of the substantia nigra pars compacta (SNc) results from interactions between intrinsic membrane conductances and afferent inputs. In adult SNc DA neurons, low-frequency tonic background activity is generated by intrinsic pacemaker mechanisms, whereas burst generation depends on intact synaptic inputs in particular the glutamatergic ones. Tonic DA release in the striatum during pacemaking is required to maintain motor activity, and burst firing evokes phasic DA release, necessary for cue-dependent learning tasks. However, it is still unknown how the firing properties of SNc DA neurons mature during postnatal development before reaching the adult state. We studied the postnatal developmental profile of spontaneous and evoked AMPA and NMDA (*N*-Methyl-D-aspartic acid) receptor-mediated excitatory postsynaptic currents (EPSCs) in SNc DA neurons in brain slices from immature (postnatal days P4–P10) and young adult (P30–P50) tyrosine hydroxylase (TH)-green fluorescent protein mice. We found that somato-dendritic fields of SNc DA neurons are already mature at P4–P10. In contrast, spontaneous glutamatergic EPSCs show a developmental sequence. Spontaneous NMDA EPSCs in particular are larger and more frequent in immature SNc DA neurons than in young adult ones and have a bursty pattern. They are mediated by GluN2B and GluN2D subunit-containing NMDA receptors. The latter generate long-lasting, DQP 1105-sensitive, spontaneous EPSCs, which are transiently recorded during this early period. Due to high NMDA activity, immature SNc DA neurons generate large and long lasting NMDA receptor-dependent (APV-sensitive) bursts in response to the stimulation of the subthalamic nucleus. We conclude that the transient high NMDA activity allows calcium influx into the dendrites of developing SNc DA neurons.

## Introduction

The dopaminergic (DA) neurons of the substantia nigra pars compacta (SNc, A9) are involved in the control of reward-related behavior and motor performances (Schultz, 2013). The spontaneous activity pattern of adult SNc DA neurons results from the interactions between intrinsic and synaptic currents. In adult rodents *in vivo*, this varies between a slow, intrinsically generated, pacemaker-like firing pattern and an irregular pattern with bursts triggered by the transient interaction between ionotropic glutamate receptors and voltage gated ionic channels (Bunney et al., 1973; Grace and Bunney, 1984a,b; Grace and Onn, 1989; Hyland et al., 2002; Fa et al., 2003; Deister et al., 2009). *In vitro*, only the pacemaker-like pattern is observed (Grace and Onn, 1989; Yung et al., 1991; Richards et al., 1997; Gulacsi et al., 2003; Paladini and Roeper, 2014), except in slices from juvenile (P15–P21) rats, where a small proportion of SNc neurons show bursting activity *in vitro* (Mereu et al., 1997).

The firing rate and firing pattern of SNc neurons are major determinants of the levels of dopamine released from terminals. The robustness of pacemaking underlies its biological importance in delivering a constant basal level of DA in target basal ganglia nuclei such as the striatum, to provide a general motivating function (Wise, 2004). In contrast, burst firing greatly and transiently increases dopamine release (Gonon, 1988; Manley et al., 1992; Chergui et al., 1994; Grillner and Mercuri, 2002), which in turn promotes the corticostriatal plasticity necessary for habit-learning (Reynolds and Wickens, 2000; Centonze et al., 2001; Calabresi et al., 2007; Kreitzer and Malenka, 2008; Schultz, 2013).

Interaction between glutamatergic afferents and ionotropic glutamate receptors play a major role in the generation of bursts in adult SNc DA neurons. Dendritic application of glutamate or NMDA (*N*-Methyl-D-aspartic acid) *in vitro* or stimulation of afferents at 50–100 Hz (but not 10 Hz) in the continuous presence of GABA_A_, GABA_B_, and D_2_ receptor antagonists, generate *in vivo*-like burst firing (Grace and Bunney, 1983; Johnson et al., 1992; Smith and Grace, 1992; Chergui et al., 1993; Christoffersen and Meltzer, 1995; Meltzer et al., 1997; Blythe et al., 2007, 2009; Wang et al., 2011). Furthermore, SNc DA bursts triggered by electrical stimulation of glutamatergic afferents or iontophoretic application of glutamate/aspartate in brain slices are blocked by NMDA receptor antagonists (Deister et al., 2009). Accordingly, genetic inactivation of NMDA receptors selectively in SNc DA neurons impairs spontaneous and evoked burst firing without affecting pacemaker-like activity (Zweifel et al., 2009). In juvenile rats, both AMPA (α-amino-3-hydroxy-5-methyl-4-isoxazolepropionic acid) and NMDA receptors are critical for transient burst-like, high-frequency firing (Blythe et al., 2007). Juvenile (P7–P28) rodent SNc neurons express AMPA-evoked currents mediated by GluA2-containing AMPA receptors, kainate (KA)-evoked currents mediated by GluK3 and GluK5-containing KA receptors and NMDA-evoked currents mediated by GluN2B- and GluN2D-containing (but not GluN2A-containing) NMDA receptors (Mereu et al., 1991; Bischoff et al., 1997; Gotz et al., 1997; Wullner et al., 1997; Counihan et al., 1998; Chatha et al., 2000; Lin and Lipski, 2001; Vivo et al., 2002; Jones and Gibb, 2005; Brothwell et al., 2008; Suarez et al., 2010). AMPA and NMDA receptors are selectively localized to a subpopulation of asymmetric synapses in the adult SNc and the two receptor types, at least partially, co-localize at individual synapses (Chatha et al., 2000).

Glutamatergic afferents to SNc neurons mainly originate in the subthalamic (STN) and pedunculopontine (PPN) nuclei. STN and PPN axons form asymmetric synapses on medium-sized and small SNc dendrites. The PPN boutons tend to form synapses onto larger diameter dendrites than the STN boutons (Hammond et al., 1978; Jackson and Crossman, 1983; Scarnati et al., 1984; Rye et al., 1987; Rinvik and Ottersen, 1993; Futami et al., 1995; Takakusaki et al., 1996).

Since AMPA and NMDA receptor activation generates transient high-frequency activity in SNc DA neurons, plasticity in their number, distribution and/or subunit composition during development could modify excitatory synaptic integration in SNc DA neurons, and thus their activity pattern later in adulthood. We therefore investigated the postnatal development of synaptic AMPA and NMDA receptor-mediated currents and how they influence the ability of SNc neurons to generate bursts in response to STN stimulation.

## Materials and Methods

All experiments were approved by the Institut National de la Santé et de la Recherche Médicale (INSERM) animal care and use agreement (D-13-055-19) and the European Community Council Directive (2010/63/UE). Animals had access to food and water ad libitum and were housed in our institutional animal facilities under a 12 h light/dark cycle at 22–24∘C.

### Mice

We used mice expressing the green fluorescent protein (GFP) under the control of the promoter of tyrosine hydroxylase (TH-GFP mice). TH-GFP mice (C57BL/6 strain; Matsushita et al., 2002) were initially backcrossed on a 129 sv background and then interbred with wild-type 129 sv mice to generate the study population of heterozygous TH-GFP mice. At P2–P5, TH-GFP pups were easily differentiated via a UV lamp from wild-type pups, because they have a fluorescent navel. Immunocytochemistry for TH in nigral slices from THGFP mice showed that only 4% of GFP-positive SNc neurons were TH-negative (**Supplementary Figure S1**).

### Slice Preparation

Mice of either sex were killed at age P4–P10 and P30–P50 (P30+) by decapitation under halothane anesthesia. Coronal or sagittal slices (400 μm thick) were cut in ice-cold oxygenated solution containing (in mM): 110 choline, 2.5 KCl, 1.25 NaH_2_PO_4_, 7 MgCl_2_, 0.5 CaCl_2_, 25 NaHCO_3_, seven glucose. During the recovery period, slices were placed at room temperature in standard artificial CSF (ACSF) saturated with 95% O_2_/5% CO_2_ and containing (in mM): 126 NaCl, 3.5 KCl, 1.2 NaH_2_PO_4_, 1.3 MgCl_2_, 2 CaCl_2_, 25 NaHCO_3_, 11 glucose.

### Electrophysiology

We recorded the activity of SNc TH-GFP neurons in the SNc dorsal to SN reticulate. We used two groups of mice: immature (P4–P10) and young adults (P30–P50). All recordings were made at 32∘C. Cells were visualized with infrared-differential interference optics (Axioskop2, Zeiss). For whole-cell voltage-clamp recordings of AMPA EPSCs and spikes, the pipette (6–10 MΩ) contained (in mM): 128.5 K-gluconate, 11.5 KCl, 1 CaCl_2_, 10 EGTA, 10 HEPES, 2.5 MgATP, 0.3 NaGTP, pH 7.32, 280 mOsm and for the recording of NMDA EPSCs, the pipette contained (in mM): 120 Cs-gluconate, 13 CsCl, 1 CaCl_2_, 10 HEPES, 10 EGTA, pH 7.2–7.4, 275–285 mOsm. Biocytin (Sigma, 5 mg/ml) was added to the pipette solution and osmolarity was corrected when necessary. We performed patch-clamp recordings in whole-cell configuration using the Digidata 1344A interface, the Multiclamp 700 A amplifier, and PClamp8 software (Molecular Devices). Access resistance ranged between 10 and 30 MΩ, and the results were discarded if access resistance changed by >20%. Since 4% of GFP-positive SNc neurons appeared to be TH-negative, we identified SNc DA according to several criteria. During recordings we identified them from their location at the dorsal edge of SN, their GFP fluorescence and their typical electrophysiological characteristics (in K gluconate recordings), i.e., action potentials of long duration (>2 ms) and a pronounced sag in response to hyperpolarizing steps. After recordings we checked the location of the recorded neurons in the SNc and their morphological characteristics (see Cell Labeling and TH Immunocytochemistry). We measured Ih amplitude by subtracting the amplitude of the current at the end of the 800 ms hyperpolarizing step to –140 mV (*V*_H_ = –60 mV) from the amplitude of the current 15 ms after the first capacitative current.

We measured spontaneous AMPA/KA currents in voltage-clamp mode at *V*_H_ = –60 mV in the continuous presence of Gabazine (5 μM) to block GABA_A_ receptors. We measured spontaneous NMDA currents in voltage-clamp mode at *V*_H_ = +40 mV in the continuous presence of Gabazine (5 μM) and NBQX (2,3-dihydroxy-6-nitro-7-sulfamoyl-benzo (f)quinoxaline-2,3-dione, 10 μM) to block GABA_A_ and AMPA/KA receptors, respectively. These currents were stored on a computer using Pclamp8 software (Molecular Devices) and analyzed off-line with Mini Analysis software (Synaptosoft 6.0), to determine the inter-event intervals (IEIs), amplitude, rise time, and decay time of spontaneous currents. The decay of spontaneous synaptic currents was well fitted by a single-exponential function, starting at the peak of the current to the time point when the current had decayed to 99.9% of its peak amplitude. All detected currents were then visually inspected to reject artifactual events. To quantify the respective contribution of AMPA receptors and KA receptors to synaptic transmission, we performed experiments in the presence of 1 μM NBQX at a dose which preferentially antagonizes AMPA receptors. NMDA spontaneous EPSCs (sEPSCs) occurred either as single events or in bursts. We defined a burst of NMDA sEPSCs as the occurrence of at least three superimposed NMDA sEPSCs and a bursty pattern as at least two bursts/cell/3 min. Miniature AMPA or NMDA currents, recorded in the presence of TTX (1 μM), were not studied because they had an extremely low frequency at all ages tested.

### STN Stimulation

We performed stimulation of the STN and whole-cell recordings of glutamatergic post-synaptic responses (EPSPs and spikes) from SNc DA neurons in current clamp mode, in medial sagittal slices, where projections from STN to SNc neurons are functional (Falkenburger et al., 2001). We delivered rectangular pulses of fixed duration (200 μs) and 100–600 μA amplitude at a frequency of 0.01–50 Hz (Grass stimulator) between the two poles of an electrode made from twisted Formvar insulated nichrom wires (66 μm diameter each, A–M Systems) positioned in the middle of the STN (only the very tips of the wires were not insulated). Postsynaptic responses evoked in SNc DA neurons by high frequency (50 Hz) stimulation of the STN in the continuous presence of gabazine (5 μM) were defined as bursts of spikes when they consisted of a depolarization giving rise to a train of action potentials that outlasted the depolarizing step. The duration of spikes (half width) was measured at half maximal amplitude.

### Drugs

Drugs were prepared as concentrated stock solutions and diluted in ACSF for bath application: gabazine, a GABA_A_ receptor antagonist, and APV (D-(-)-2-amino-5-phosphonopentanoic acid), an NMDA receptor antagonist and tetrodotoxin (TTX) a blocker of voltage-gated Na^+^ channels were purchased from Sigma. NBQX, an AMPA/KA receptor antagonist and was donated by the National Institute of Mental Health, NIMH chemical synthesis and drug supply program. DQP 1105 [5-(4-Bromophenyl)-3-(1,2-dihydro-6-methyl-2-oxo-4-phenyl-3-quinolinyl)-4,5-dihydro-g-oxo-1H-pyrazole-1-butanoic acid] (10 μM), a non-competitive NMDA receptor antagonist which displays over 50-fold selectivity for GluN2D- and GluN2C-containing receptors over GluN2B- containing receptors, and Ro 25-6981 maleate (αR,βS)-α-(4-Hydroxyphenyl)-β-methyl-4-(phenylmethyl)-1-piperidinepropanol maleate) (1 μM), a selective activity-dependent blocker of NMDA receptors containing the GluN2B subunit, were both purchased from Tocris.

### Cell Labeling and TH Immunocytochemistry

After the recording session, to localize and analyze the recorded SNc DA neurons, we visualized the biocytin-injected neurons. After 24 h in paraformaldehyde (3%) at 4∘C, the sections were rinsed in PBS and preincubated for 1 h in 0.3% Triton X-100 (Abcys) in PBS with 5% normal goat serum (NGS) at room temperature. Slices were then incubated in Streptavidin-Cy3 (1:500) in PBS-Triton X-100 (0.3%) and NGS (5%) for 12 h at 4∘C. After thorough rinsing, slices were mounted in Fluoromount and coverslipped. Confocal Images were taken with a Leica SP5-X (objective ×40, NA = 1.3) and stacks were filtered (median 3D) and stitched (3D stitching) with FIJI software (ImageJA v1.46b, Open source software, http://pacific.mpi-cbg.de). Dendritic and axonal arbors were reconstructed for morphological analysis using the Neurolucida system (MicroBrightField Inc.). To check whether GFP expression was restricted to TH-positive neurons of the SNc, slices were incubated in anti-TH polyclonal antibody (Pel-Freez, dilution of 1:1000) for 12 h. After rinsing with PBS, slices were incubated in anti-rabbit IgG secondary antibody Alexa A555 (Life Technologies, dilution of 1:500) for 1 h. To count TH-positive and GFP-positive neurons, images were taken with a Zeiss observer Z1 microscope with Apotome device (structured illumination), 20× magnification.

### Statistics

Results are given in the text and figures in the form of mean ± SEM. Non-parametric Mann–Whitney test (Graphpad Prism 5 software, San Diego, CA, USA) was used to compare results from P4–P10 and P30+ SNc DA neurons. Since there was no significant difference between the full set of results obtained at P4–P5 and at P8–P10 for AMPA- and NMDA-mediated sEPSCs (data not shown), we pooled the results obtained between P4 and P10. For example, there was no significant difference for the frequency (*P* = 0.3), and amplitude (*P* = 0.5) of AMPA sEPSCs. Similarly, there was no significant difference for the frequency (*P* = 0.35), amplitude (*P* = 0.52) of single NMDA sEPSCs, nor for the characteristics of bursts of NMDA sEPSCs: burst frequency (*P* = 0.34), burst duration involving at least three events (*P* = 0.15), and mean intraburst frequency (*P* = 0.15). A non- parametric two-tailed paired *t*-test was used to compare values obtained at a given age before and during drug application. Kolmogorov–Smirnov test was used to compare cumulative distributions. For each test performed the P value was provided and the statistical significance was taken at *P* ≤ 0.05.

## Results

### Developmental Characteristics of Dendritic Field and Intrinsic Membrane Properties of SNc DA Neurons

**FIGURE 1:**
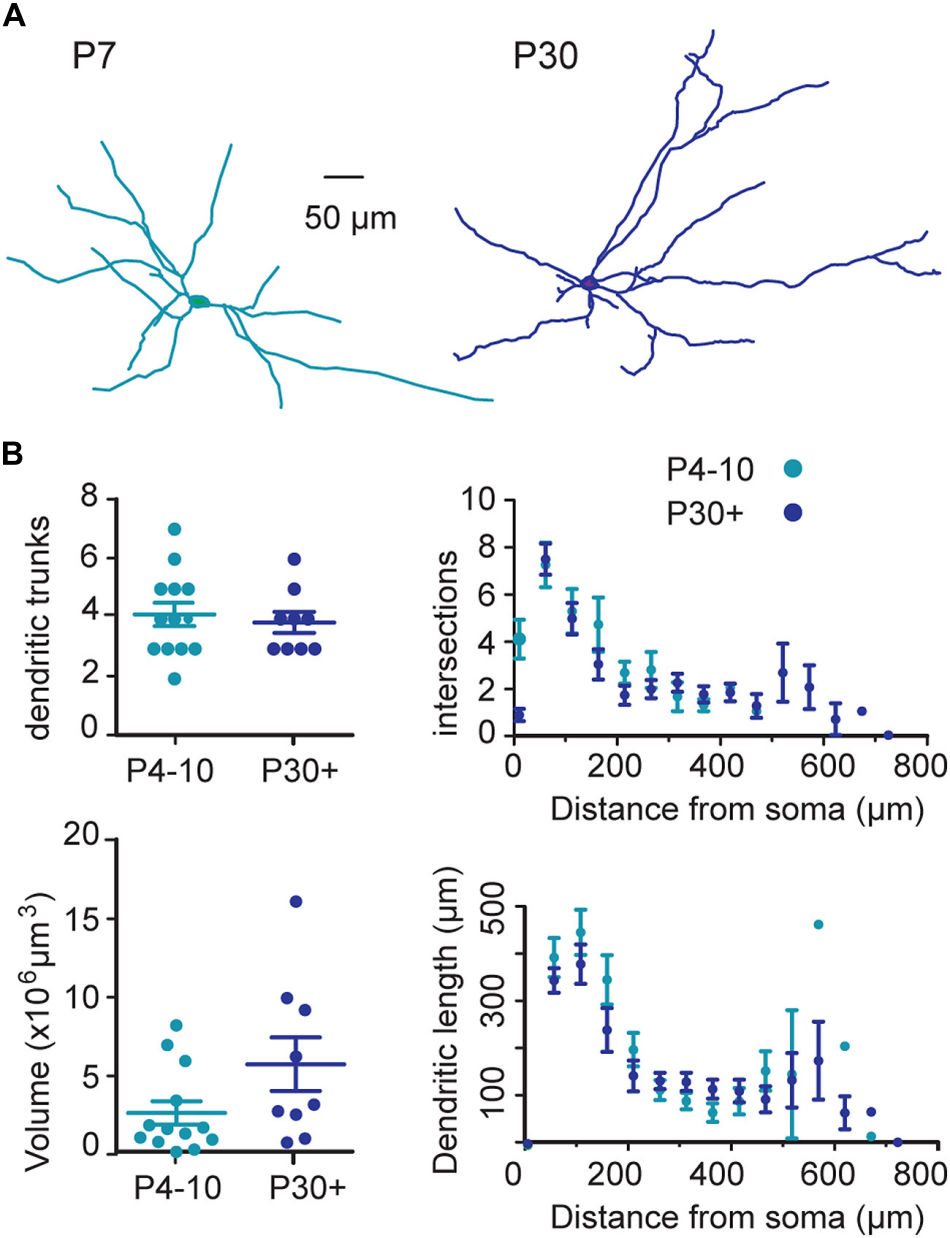
**Morphological properties of somato-dendritic fields of substantia nigra pars compacta (SNc) dopaminergic (DA) neurons during postnatal development. (A)** Typical examples of neurolucida reconstruction of biocytin-filled SNc tyrosine hydroxylase (TH)-green fluorescent protein (GFP) neurons at the indicated ages. **(B)** Quantification and statistical comparison between P4–P10 and P30+ of the number of dendritic trunks (top left), of the convex hull volume occupied by the dendritic trees (bottom left), and of the extent of the dendritic trees (3D Scholl analysis) (top and bottom right). In this (**B**, left) and following figures, each dot represents one cell, the distance between bottom and top bars represents the SEM, and the middle bar indicates the mean value.

The dendritic field of SNc DA neurons did not significantly change between P4–P10 (*n* = 13) and P30+ (*n* = 9) (**Figure 1**). The mean number of dendritic trunks was 4 ± 1 at P4–P10 and 4 ± 1 at P30+ (*P* = 0.7), the mean number of dendritic ends was 13 ± 1 at P4–P10 and 12 ± 1 at P30+ (*P* = 0.6), the mean total dendritic length was 1386 ± 202 μm at P4–P10 and 1522 ± 249 at P30+ (*P* = 0.6) and the mean dendritic volume was 2.6 × 10^6^ ± 0.7 × 10^6^μm^3^ at P4–P10 and 5.7 × 10^6^± 1.7 × 10^6^ at P30 (*P* = 0.1). The axon originated either from the soma (*n* = 5/13 at P4–P10; *n* = 2/9 at P30+) or from a primary dendritic trunk (*n* = 8/13 at P4–P10; *n* = 7/9 at P30+), as already described for P15–P20 SNc DA neurons (Hausser et al., 1995; Gentet and Williams, 2007; Blythe et al., 2009).

The mean membrane resistance of P4–P10 neurons was twice as high (468.8 ± 54.5 MΩ, *n* = 38) as that of P30+ neurons (230.7 ± 18.1 MΩ, *n* = 22, *P* = 0.0002), but their mean membrane capacitance was similar for the two age groups (30.9 ± 2.9 pF and 32.3 ± 2.9 pF, respectively, *P* = 0.58). The amplitude of the inwardly rectifying H current (*I*_h_) in response to hyperpolarizing pulses to –140 mV (*V*_H_ = –60 mV), was similar at P4–P10 (–327.3 ± 34.7 pA, *n* = 32) to that at P30+ (–272.3 ± 31.5 pA, *n* = 11, *P* = 0.57).

### AMPA Receptor-Mediated sEPSCs Follow a Postnatal Developmental Sequence

We recorded sEPSCs (*V*_H_ = –60 mV) at P4–P10 and at P30+ that were insensitive to APV (40 μM), the specific antagonist of NMDA receptors, but fully blocked by the addition of NBQX 1 μM, a preferential antagonist of AMPA receptors (*n* = 6, data not shown). Application of 1 μM NBQX specifically blocked the AMPA receptor-mediated current and allowed the measurement of the KA receptor-mediated current. In this condition, we demonstrated that neither immature nor young adult SNc neurons generate spontaneous KA-receptor-mediated EPSCs.

**FIGURE 2:**
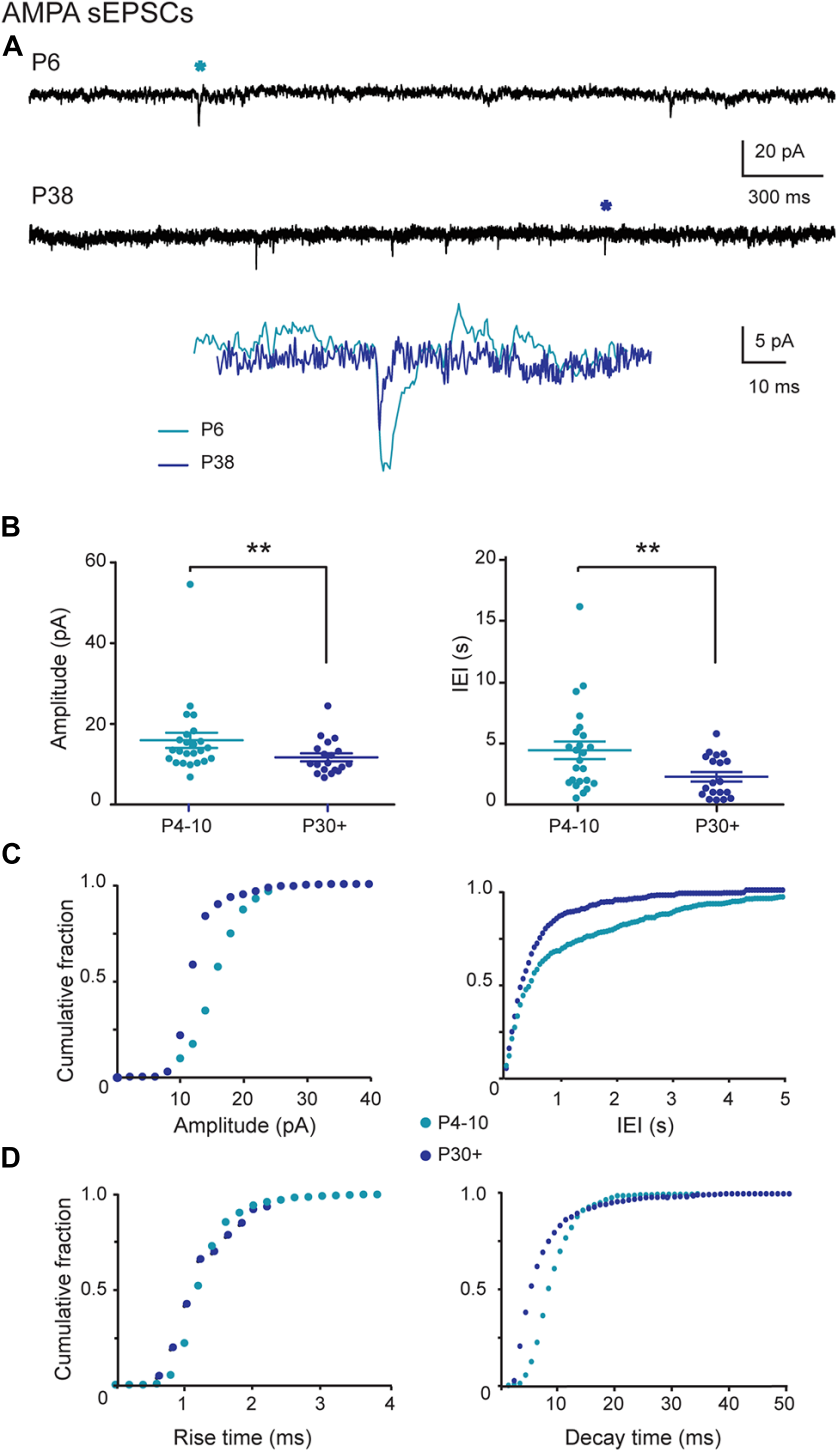
**AMPA (α-amino-3-hydroxy-5-methyl-4-isoxazolepropionic acid) sEPSCs in SNc DA neurons during postnatal development. (A)** Voltage-clamp recordings of AMPA sEPSCs from P4 and P30 midbrain TH-GFP neurons in the continuous presence of gabazine (5 μM) and APV (40 μM) at *V*_H_ = –60 mV. AMPA events indicated by a star are enlarged and superimposed in the inset. **(B)** Quantification and statistical comparison of the amplitudes and inter-event intervals (IEIs) of P4–P10 (*n* = 24) and P30+ (*n* = 19) AMPA sEPSCs. **(C)** Cumulative distributions of the amplitude and IEI of AMPA sEPSCs for the two age groups (bin width 1 pA and 50 ms). At P4–P10, 50% of single AMPA excitatory postsynaptic currents (EPSCs) had a amplitude of at most 15.9 pA and an IEI of at most 450 ms, whereas this median was 12.5 pA and 300 ms at P30+. **(D)** Cumulative distributions of the rise and decay times of AMPA sEPSCs for the two age groups (bin width 0.2 pA and 1 ms). The median rise and decay times were, respectively 1.2 and 8.0 ms at P4–P10, and 1.1 and 4.9 ms at P30+. Number of events: 1921 (P4–P10) and 1317 (P30+). In this and subsequent figures, when indicated, ^∗^*P* < 0.05 and ^∗∗^*P* < 0.01.

AMPA receptor-mediated sEPSCs (AMPA sEPSCs) had larger amplitudes (*P* = 0.017) and longer IEIs (*P* = 0.005) in immature than in young adult SNc DA neurons. The mean amplitude and mean IEI were 16.0 ± 1.9 pA and 4.4 ± 0.7 s in P4–P10 SNc neurons (*n* = 24) and 11.8 ± 1.0 pA and 2.1 ± 0.4 s in P30+ SNc neurons (*n* = 19; **Figures 2A,B**). The cumulative frequency distributions for the amplitude or the IEIs of single AMPA EPSCs also differed significantly (P = 0.018 and P < 0.001, respectively; Kolmogorov–Smirnov test) between the two age groups (**Figure 2C**). Single AMPA sEPSCs had a similar rise time at P4–P10 and at P30+ (1.17 ± 0.08 ms, *n* = 24, 1.11 ± 0.09 ms, *n* = 19, respectively; *P* = 0.6) but had a longer mean decay time at P4–P10 (9.3 ± 1.7 ms) than at P30+ (6.0 ± 1.1 ms, *P* < 0.0001; **Figure 2D**). Similarly, cumulative frequency distributions for the rise time of AMPA-mediated sEPSCs did not differ significantly between P4–P10 and P30+ (*P* > 0.99), but the decay times were significantly different (*P* < 0.0001; **Figure 2D**). Therefore, AMPA sEPSCs in SNc DA neurons mature between P4–P10 and P30+.

### NMDA Receptor-Mediated sEPSCs Follow a Postnatal Developmental Sequence

**FIGURE 3:**
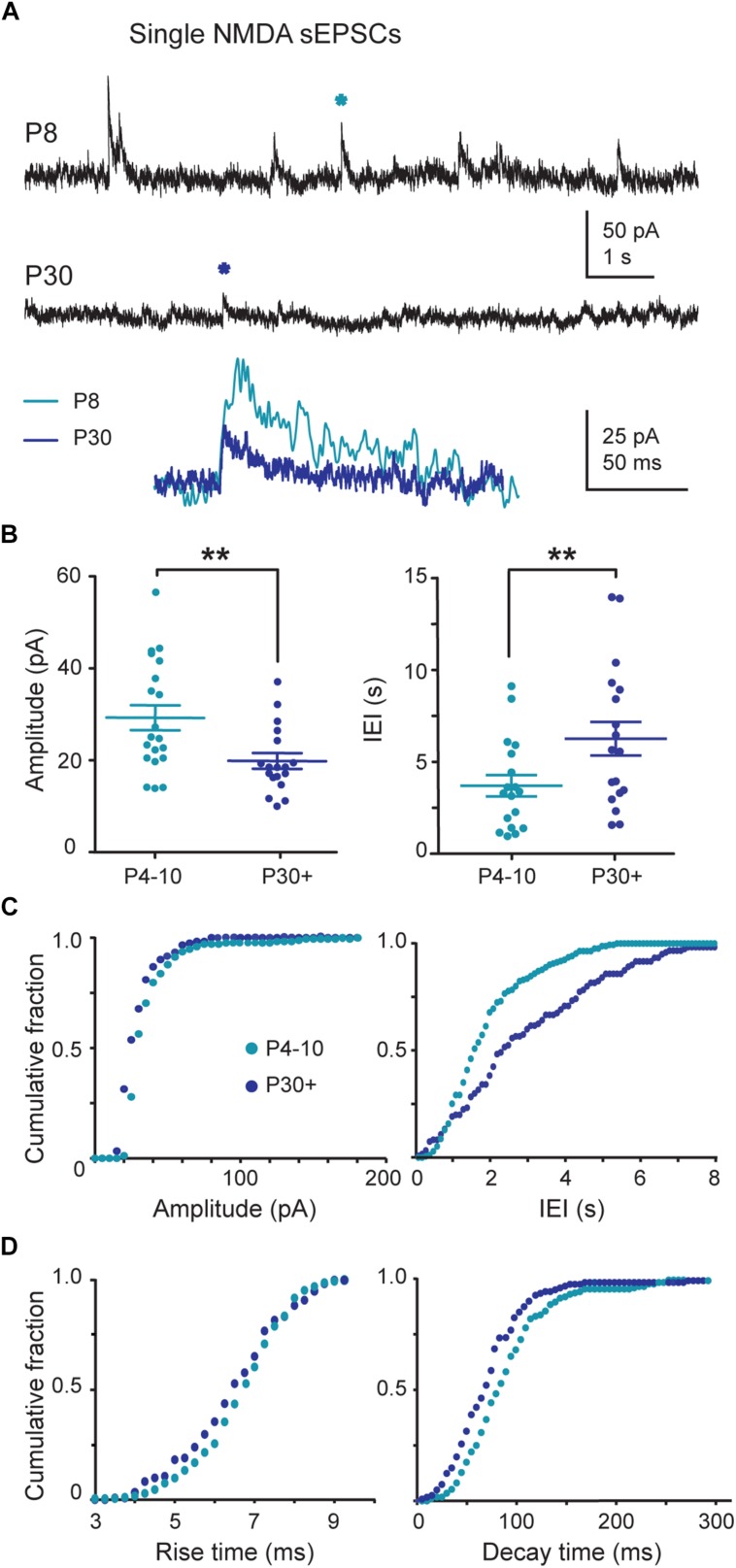
**Single NMDA (*N*-Methyl-D-aspartic acid) sEPSCs during postnatal development. (A)** Voltage-clamp recordings of NMDA sEPSCs from P8 and P30 SNc TH-GFP neurons in the continuous presence of gabazine (5 μM) and NBQX (10 μM) at *V*_H_ = +40 mV. The P8 and P30 NMDA events indicated by a star are enlarged and superimposed in the inset. **(B)** Quantification and statistical comparison of the data from P4–P10 (*n* = 20) and P30+ (*n* = 18) NMDA sEPSCs, as indicated. **(C)** Cumulative distributions of the amplitude and IEI of single NMDA sEPSCs for the two age groups (bin width 5 pA and 100 ms). At P4–P10, 50% of single NMDA EPSCs had an amplitude of at most 31.6 pA and an IEI of at most 1.6 s whereas this median was 25.8 pA and 2.4 s at P30+. **(D)** Cumulative distributions of the rise and decay times of single NMDA sEPSCs for the two age groups (bin width 0.25 and 5 ms). The median rise and decay times were respectively 6.8 and 84.6 ms at P4–P10 and 6.6 and 71.9 ms at P30+. Number of events: 1350 (P4–P10) and 770 (P30+). In this and subsequent figures, when indicated, ^∗^*P* < 0.05 and ^∗∗^*P* < 0.01.

**FIGURE 4:**
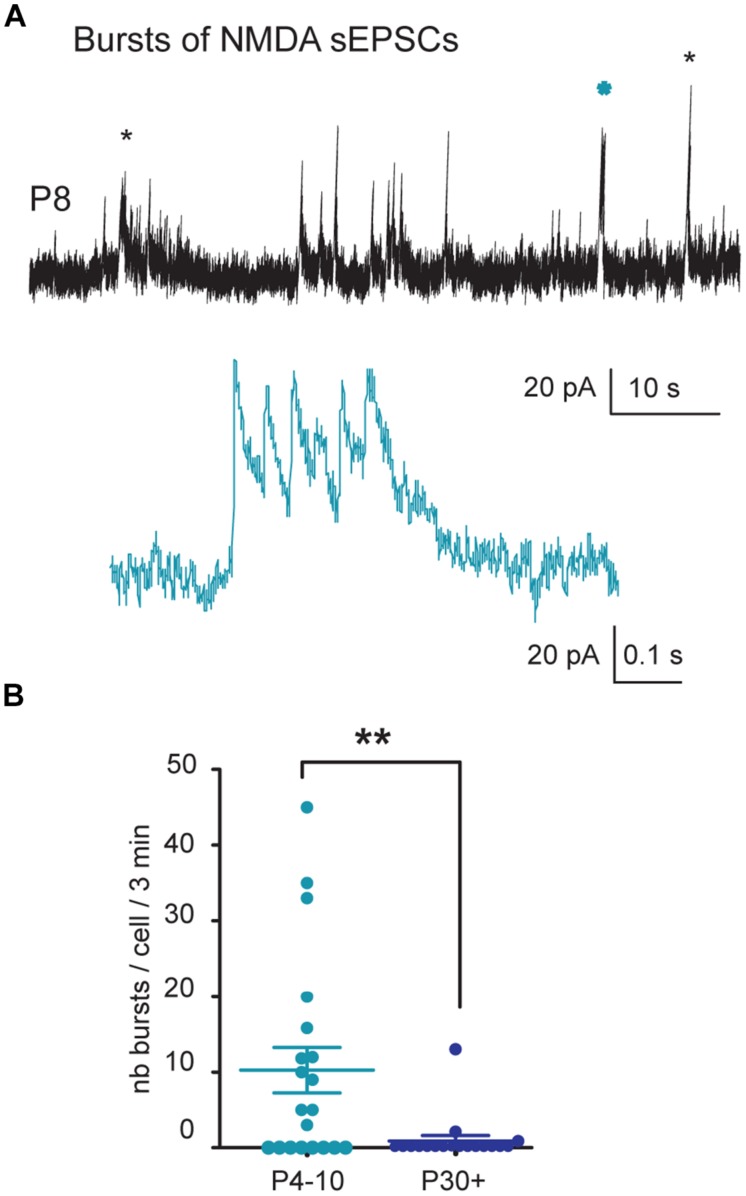
**Bursts of NMDA sEPSCs during postnatal development. (A)** Voltage-clamp recordings of NMDA sEPSCs from a P8 SNc TH-GFP neuron in the continuous presence of gabazine (5 μM) and NBQX (10 μM) at *V*_H_ = +40 mV. The stars (black and blue) indicate bursts of NMDA sEPSCs. The other events are single events either isolated or in trains. The burst indicated by a blue star is shown in the inset at a more rapid time base. **(B)** Quantification and statistical comparison of the number of bursts per cell per 3 min from P4–P10 and P30+ SNc DA neurons. In this and subsequent figures, when indicated, ^∗^*P* < 0.05 and ^∗∗^*P* < 0.01.

At P4–P10, single NMDA sEPSCs showed greater amplitude (29.3 ± 2.7 pA) and shorter IEIs (3.6 ± 0.6 s, *n* = 20 neurons) than at P30+ (amplitude 20.0 ± 1.7 pA; 6.2 ± 0.9 s, *n* = 18; *P* = 0.007 and 0.027, respectively; **Figures 3A,B**). The cumulative frequency distributions for the amplitude or the IEIs of single NMDA sEPSCs differed significantly (*P* < 0.0001 for both, Kolmogorov–Smirnov test) between the two age groups (**Figure 3C**). Single NMDA sEPSCs also showed a longer mean decay time at P4–P10 (92.6 ± 3.8 ms, *n* = 20) than at P30+ (75.5 ± 4.5 ms, *n* = 18, *P* = 0.0003) but a similar mean rise time (7.3 ± 0.2 ms and 7.6 ± 0.3 ms, *P* = 0.44). The cumulative frequency distributions also differed significantly between the two age groups with respect to decay time (*P* < 0.0001, Kolmogorov–Smirnov test), but not with respect to rise time (*P* = 0.9; **Figure 3D**). Strikingly, in the P4–P10 group but not in the P30+ one, NMDA sEPSCs often occurred in bursts. The bursty pattern was present in 60% of P4–P10 (*n* = 12/20) versus 11% (2/18) of P30+ SNc DA neurons. Moreover, P4–P10 bursts were more frequent and consisted of more events (mean burst frequency: 0.04 ± 0.01 Hz; mean burst duration: 416 ± 91 ms involving 3–21 events with a mean intraburst frequency of 17.3 ± 3.1 Hz; *n* = 146 bursts in 12 neurons) than P30+ bursts (mean burst frequency: 0.02 ± 0.01 Hz, mean burst duration: 259 ± 95 ms involving 3–5 events with a mean intraburst frequency of 19.1 ± 8.7 Hz, *n* = 15 bursts in three neurons; **Figure 4**). Statistical comparison between the two groups was rendered impossible by the very small number of bursts generated by young adult SNc DA neurons.

#### Early Bursting NMDA sEPSCs were Mediated by GluN2D-Containing NMDA Receptors

**FIGURE 5:**
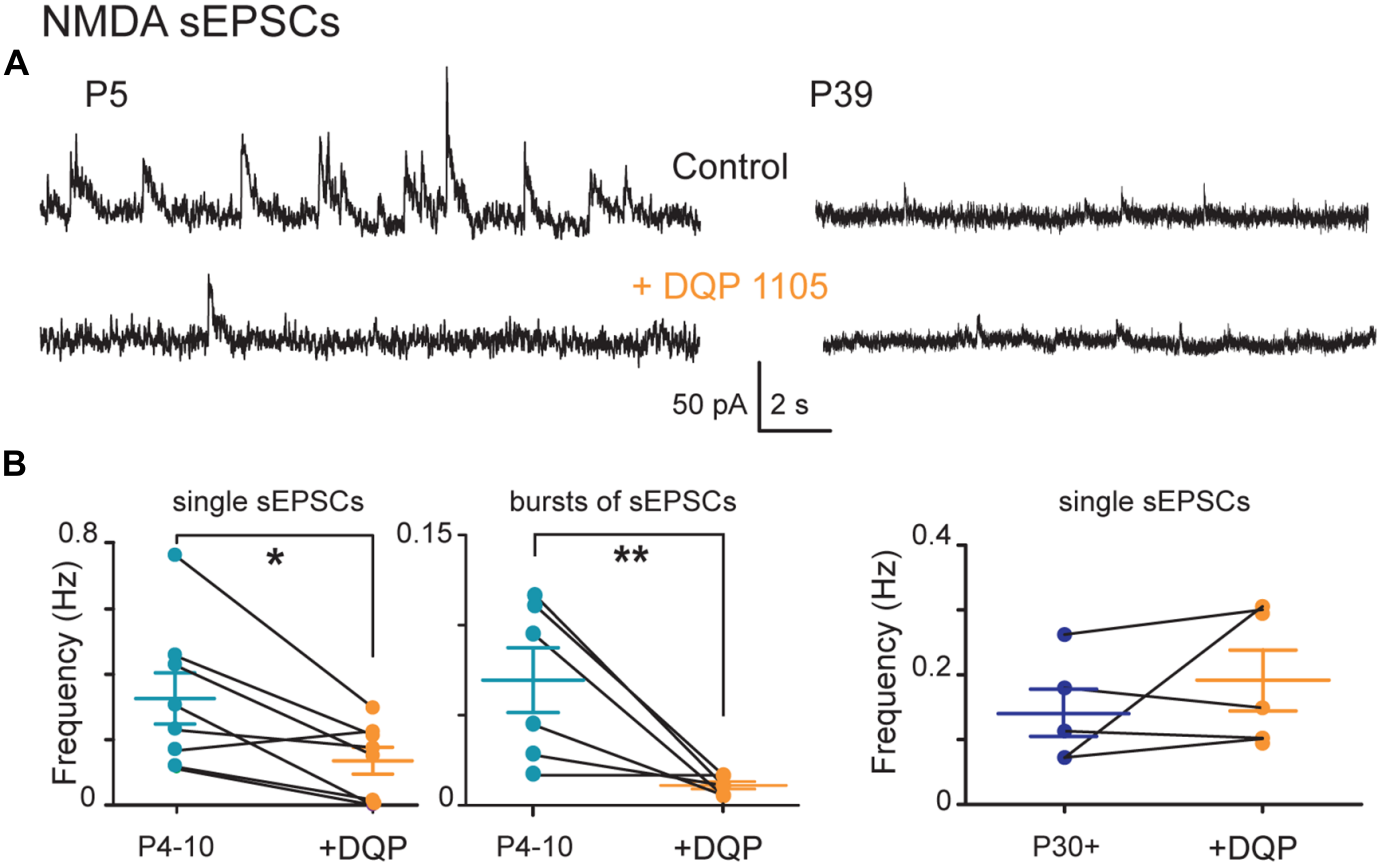
**Pharmacology of single and bursts of NMDA sEPSCs during postnatal development. (A)** Voltage-clamp recordings of NMDA sEPSCs from a P5 and a P39 SNc TH-GFP neuron in the continuous presence of gabazine (5 μM) and NBQX (10 μM) at *V*_H_ = +40 mV in the absence (top) or presence (bottom) of DQP1105 (10 μM). **(B)** From left to right, quantification of the effect of DQP 1105 on the frequency of single (left) and bursts of (right) NMDA sEPSCs recorded at P4–P10. In this and subsequent figures, when indicated, ^∗^*P* < 0.05 and ^∗∗^*P* < 0.01.

We investigated whether the developmental shift of NMDA sEPSCs was due to altered NMDA receptor subunit composition. During development SNc neurons express NMDA-evoked currents mediated by GluN2B- and GluN2D-containing (but not GluN2A-containing) NMDA receptors. We first tested DQP 1105, at a dose (10 μM) which preferentially blocks GluN2C/D-containing receptors. It decreased the frequency of P4–P10 single NMDA sEPSCs by 56% (from 0.32 ± 0.08 to 0.14 ± 0.04 Hz, *n* = 8 neurons; *P* = 0.02 non-parametric paired *t*-test) but had no effect on P30+ single NMDA sEPSCs amplitude (17.1 ± 2.3 pA before and 16.1 ± 1.7 pA during DQP, *n* = 5; *P* = 0.3) or frequency (0.14 ± 0.04 Hz before and 0.19 ± 0.05 Hz during DQP, *n* = 5; *P* = 0.2; **Figure 5**). DQP 1105 also significantly reduced the occurrence of bursts of P4–P10 NMDA sEPSCs (interburst frequency: 0.07 ± 0.02 Hz before and 0.010 ± 0.002 Hz during DQP; *P* = 0.007) but did not change either their duration (550 ± 137 ms before and 810 ± 456 ms during DQP; *P* = 0.8) or their intraburst frequency (12.0 ± 2.6 Hz before and 14 ± 3 Hz during DQP; *P* = 0.8; *n* = 6). Subsequent application of Ro 25-6981 (1 μM), a preferential blocker of GluN2B-containing NMDAR, abolished all single and bursting NMDA sEPSCs, in P4–P10 and P30+ SNc DA neurons (data not shown). Application of Ro 25-6981, before that of DQP 1105, also blocked all ongoing NMDA sEPSCs in both groups, demonstrating that all NMDA receptors contain NR2B subunits.

#### DQP 1105 had a Postsynaptic Action

To investigate whether the GluN2D-containing NMDA receptors were localized pre- or postsynaptically, we checked whether DQP 1105 (10 μM) affected the amplitude and frequency of AMPA sEPSCs in P4–P10 SNc neurons. DQP 1105 had no effect on either the mean amplitude (14.4 ± 2.6 pA before DQP vs 15.0 ± 2.9 pA after DQP, *n* = 5, *P* = 1) or the mean frequency (1.8 ± 0.8 Hz before DQP vs 2.1 ± 0.9 Hz after DQP, *n* = 5, *P* = 0.19) of AMPA sEPSCs of immature SNc neurons (data not shown). Taken together, our results demonstrate that GluN2D-containing NMDA receptors are transiently expressed at P4–P10 in SNc DA neurons and are expressed postsynaptically.

All the above results indicate that a GluN2B subunit is present in NMDARs of both immature and young adult SNc DA neurons and is indispensable for the generation of NMDA sEPSCs. In contrast, the GluN2D-containing NMDA receptor follows a developmental sequence responsible for the generation of large and frequent single sEPSCs and bursts of sEPSCs in immature SNc DA neurons. Subsequently, GluN2D subunit-containing NMDA receptor-mediated sEPSCs disappear in young adult SNc DA neurons, probably due to the disappearance of GluN2D subunit-containing receptors.

### The AMPA/NMDA Ratio of Glutamatergic Receptor-Mediated EPSCs Follows a Developmental Sequence

**FIGURE 6:**
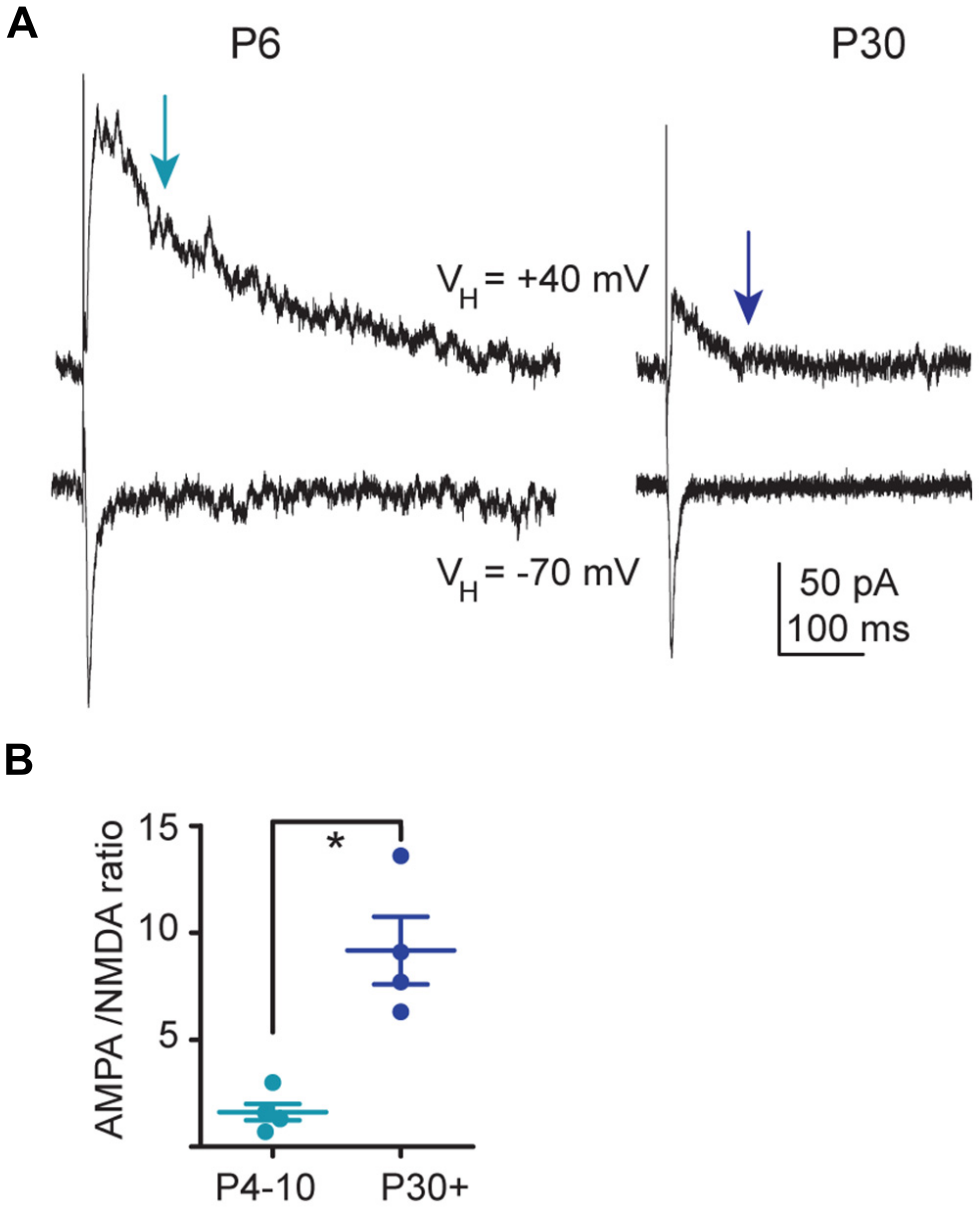
**AMPA/NMDA ratio during development. (A)** Voltage clamp recordings in the same SNc TH-GFP neurons of the subthalamic nucleus (STN)-evoked AMPA EPSC (bottom traces, *V*_H_ = –70 mV) and of the STN-evoked AMPA–NMDA EPSC (top traces, *V*_H_ = +40 mV) at P6 (left) and P30 (right) in the continuous presence of gabazine (5 μM). **(B)** Statistical comparison of the ratios of the peak amplitude of the AMPA EPSC to the amplitude of the NMDA EPSC measured at *t* = 100 ms after the artifact (red arrow in **A**), as a function of age. In this and subsequent figures, when indicated, ^∗^*P* < 0.05.

We investigated whether a change in NMDA current during development would be evidenced by a change in AMPA/NMDA ratio. Comparison of the STN-evoked monosynaptic responses at *V*_H_ = –70 mV and +40 mV, before and after bath application of APV (40 μM), showed that at *V*_H_ = –70 mV the inward EPSC was purely mediated by AMPAR and at *V*_H_ = +40 mV, 100 ms after its onset, the outward EPSC was purely mediated by NMDAR (not shown). To obtain the AMPA/NMDA ratio, we calculated, in the each recorded neuron, the ratio of the peak amplitude of the STN-evoked inward EPSC at *V*_H_= –70 mV and of the amplitude of the evoked outward EPSC at +40 mV, 100 ms after the EPSC onset. This gave a lower AMPA/NMDA ratio (1.6 ± 0.3, *n* = 13) at P4–P10 than at P30+ (10.5 ± 2.8, *n* = 11, *P* = 0.0001). Therefore, NMDA currents largely predominate in P4–P10 SNc DA neurons, in contrast to P30+ (**Figure 6**).

### STN-Evoked Burst Firing of SNc DA Neurons Follows a Developmental Sequence

**FIGURE 7:**
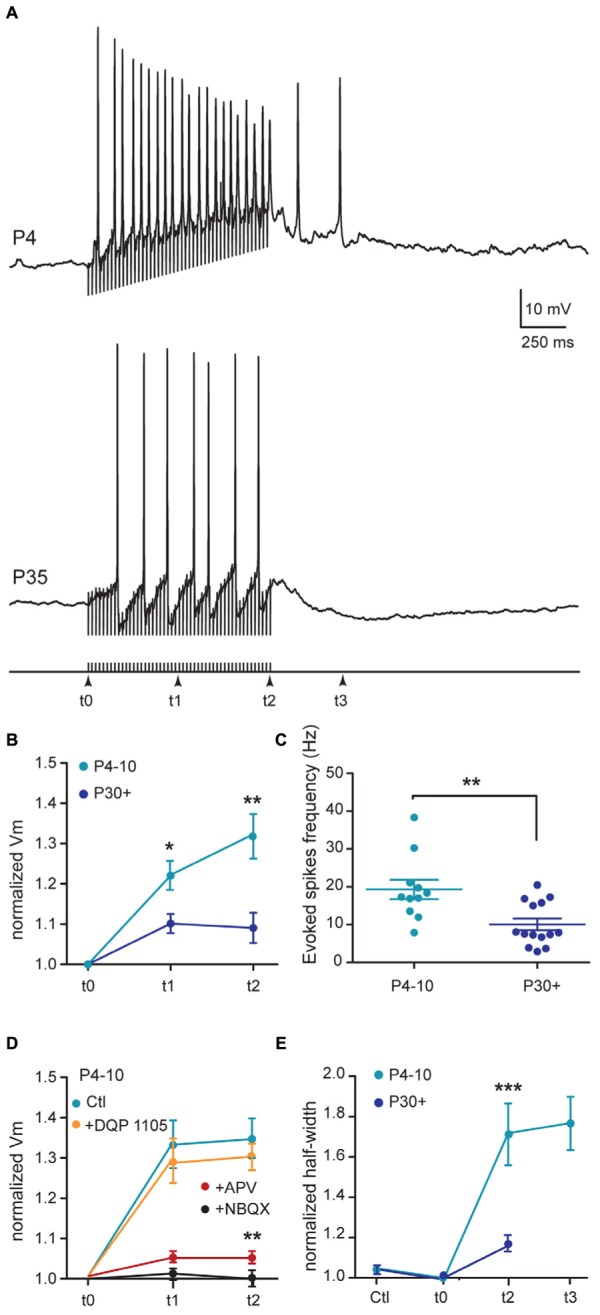
**Subthalamic nucleus-evoked responses of SNc DA neurons during development. (A)** Typical examples of current clamp recordings (*V*_m_ = –43/–45 mV) of responses of SNc DA neurons to 1 s STN stimulation (200 μs stimuli at 50 Hz) in the continuous presence of gabazine (5 μM), at the indicated ages. **(B)** Quantification and statistical comparison of the percent of membrane depolarization (% Δ*V*_m_) measured at three different times during the 50 Hz stimulation as indicated in **(A)** bottom, in P4–P10 and P30+ neurons. **(C)** Spike frequency during the stimulation period. **(D)** Normalized membrane depolarization (*V*_m_) measured at three different times during the 50 Hz stimulation in the absence (control) and in the presence of cumulative applications of NMDAR and AMPAR blockers, as indicated (P4–P10 SNc DA neurons). **(E)** Half-width of the spike just before STN stimulation (tCtl), of the last spike evoked during the stimulation period (t2) and of the last spike evoked during the after-depolarization (t3), normalized to the half-width of the first spike at the onset of the stimulation period (t0). Spikes at t3 are absent from P30+ recordings due to the absence of after-depolarization at that age. The stars in **(B,C,E)**, indicate the statistical results between P4–P10 and P30+. The stars in **(D)** indicate the statistical results between control (Ctl) and APV, for the same age group (P4–P10). In this and subsequent figures, when indicated, ^∗^*P* < 0.05, ^∗∗^*P* < 0.01, and ^∗∗∗^*P* < 0.001.

We stimulated STN neurons at 50 Hz (1 s duration) in sagittal slices to study the ability of SNc DA neurons to generate bursts of action potentials (Blythe et al., 2007; Hage and Khaliq, 2015). In the continuous presence of GABA_A_ receptor antagonists, STN stimulation evoked long-lasting bursts of spikes in juvenile SNc DA neurons but not in young adult ones. At P4–P10, before stimulation, control spike frequency was 1.58 ± 0.46 Hz (*V*_m_ = –42.2 ± 1.3 mV). The 50 Hz STN stimulation depolarized the membrane by 30% (of 12.6 ± 2.6 mV, measured at the end of the 1 s stimulation), and generated spikes at 16.7 ± 1.7 Hz (*n* = 16; **Figures 7A–C**). At the end of the 1 s stimulation the amplitude of the evoked action potentials was 33% smaller than that of the first spike in the train (from 63.3 ± 2.4 to 40.4 ± 3.6 mV, *P* = 0.0006) and their half-width 76% greater (from 3.4 ± 0.3 to 6.2 ± 0.7 ms, *P* < 0.0001; **Figures 7A,E**). The STN-evoked depolarization was followed by an after- depolarization which outlasted the 1s stimulation period by 623 ± 174 ms and generated spikes at 5.1 ± 1.6 Hz (*n* = 12/16; **Figure 7A**). Bath application of DQP (10 μM) had no significant effect on the depolarization amplitude of the evoked burst (*n* = 7, *P* > 0.99; **Figure 7D**), nor on spike frequency during the stimulation (*P* = 0.7) or during the after- depolarization (*P* = 0.8). Subsequent application of APV (40 μM) reduced the amplitude of the STN-evoked depolarization by 78% (from 11.5 ± 1.9 to 2.0 ± 0.8 mV, measured at the end of the stimulation period, *P* = 0.001), reduced spike frequency during the stimulation by 66% (*P* = 0.02) and totally abolished the after-depolarization. Subsequent application of NBQX (10 μM) totally abolished the APV-insensitive depolarization (**Figure 7D**). At P30+ (*V*_m_ = –45.2 ± 1.4 mV), the spike frequency before stimulation was 0.97 ± 0.17 Hz (not significantly different from that at P4–P10, *P* = 0.21). The 50 Hz STN stimulation depolarized the membrane by only 9% (3.9 ± 1.7 mV) and generated spikes at 10.1 ± 1.6 Hz (**Figures 7A,B**). The evoked depolarization never outlasted the 1 s stimulation (*n* = 14/14). All the above results suggest that STN stimulation evoked a larger NMDA-dependent depolarization at P4–P10 than at P30+ (*P* = 0.007), with a longer duration due to the presence of after-depolarization, as well as evoking spikes at a higher frequency at P4–P10 than at P30+ (*P* = 0.005).

## Discussion

We show here that spontaneous AMPAR-mediated and NMDAR-mediated currents generated by SNc DA neurons mature over the first postnatal month. In particular, NMDA sEPSCs follow a characteristic developmental sequence. In immature compared to mature SNc neurons, NMDA sEPSCs have a greater amplitude and frequency, coupled with a characteristic bursty pattern, correlated with the transient expression of the slowly decaying, DQP 1105-sensitive, sEPSCs mediated by GluN2D subunit-containing NMDA receptors. STN-evoked bursts of spikes were also much larger in immature SNc DA neurons due to the presence of a high NMDAR-mediated component. This high NMDA activity of immature SNc DA neurons may allow the integration of synaptic inputs over longer periods, with larger calcium influxes into developing dendrites.

The dendritic tree of SNc DA neurons is already mature just after birth. In our earlier study, we found that midbrain DA neurons at late embryonic (E16–E18) and perinatal (P0–P1) stages (Ferrari et al., 2012) extend long axons that already reach the striatum at embryonic day E16, before developing their dendritic tree. Then the dendritic tree progressively matures from E16 (90 ± 31 μm total dendritic length; 1.5 ± 0.4 ends; *n* = 13) to P0 (1031 ± 287 μm; 12 ± 3; *n* = 6, *P* = 0.0001). When dendritic trees at P0 (Ferrari et al., 2012) were compared with dendritic trees at P4–P10 (the present study), no further maturation of dendritic length was observed (*P* = 0.09 Mann–Whitney test; compare **Figure 1A** in Ferrari et al., 2012 and **Figure 1B**). In conclusion, our results suggest that maturation of the morphological properties of the SNc DA neurons occurs during the very early prenatal period, also in agreement with previous results in the rat, where the morphology of TH positive neurons at P1 appears essentially the same as in adults (Tepper et al., 1994; Park et al., 2000).

The absence of spontaneous KAR-mediated currents in young and juvenile SNc DA neurons found here, contrasts with the large inward current previously evoked in outside-out patches of P10–P15 SNc DA neurons by bath application of KA (1 mM; Gotz et al., 1997). This suggests that the GluK1 (GluR5), GluK3 (GluR7), and GluK5 (KA2) receptor subtypes identified in rodent SNc (Bischoff et al., 1997; Wullner et al., 1997) are not activated by spontaneous release of glutamate in slices.

AMPA sEPSCs mature during the first postnatal month and display larger amplitudes in immature than in young adult SNc DA neurons. AMPAR expressed in juvenile SNc DA neurons are mainly GluA2-containing (calcium impermeable) receptors as shown by the I-V relationships in Na^+^- or Ca^2+^-enriched extracellular media (Gotz et al., 1997). Therefore synaptic activation of AMPAR is not expected to produce Ca^2+^ inflow in SNc DA neurons.

The major change during the first postnatal month concerns NMDA activity. The striking characteristic is the high spontaneous NMDA activity at P4–P10, as evidenced by large and frequent single NMDA events, sometimes with a bursty pattern, and the large APV-sensitive bursts of spikes evoked in immature but not in young adult SNc DA neurons by STN stimulation.

The spontaneous NMDA EPSCs are recorded in coronal slices where the somas of STN or PPN neurons that send glutamatergic projections to SNc DA neurons are absent. The only glutamatergic neurons [positive for the vesicular glutamate transporter type 2 (VGLuT2) mRNA] present in nigral coronal slices and that may send projections to SNc DA neurons are those described in SNc (A9) and the ventral tegmental area (VTA, A10). In contrast to VTA neurons which co-express VGluT2 mRNA and TH in a subgroup of neurons, the VGluT2 neurons in the SNc lack TH (Yamaguchi et al., 2007, 2011, 2013). This suggests that SNc and/or VTA glutamatergic neurons may connect to SNc DA neurons. Bursts of NMDA sEPSCs are likely to result from the summation of the slowly decaying NMDA sEPSCs generated by the transiently expressed, DQP 1105-sensitive, GluN2D-containing NMDA receptors (Dunah et al., 1998; Acker et al., 2011). GluN2D mRNA and protein, but not GluN2C, are preferentially expressed in the mesencephalon (Brothwell et al., 2008). Therefore the DQP-sensitive NMDA sEPSCs recorded here were mainly mediated by GluN2D subunit-containing NMDA receptors which have the slowest decay time. The deactivation time constant for the macroscopic current mediated by GluN1/GluN2D assemblies is 6–10 times slower than GluN1/GluN2B (Monyer et al., 1994; Vicini et al., 1998; Wyllie et al., 1998). This characteristic allows the summation of NMDA sEPSCs and the generation of bursts of sEPSCs. We propose that at P4–P10, NMDA sEPSCs result from the activation of diheteromeric GluN1/GluN2B receptors, together with triheteromeric GluN1/GluN2B/GluN2D receptors, (as already described in P14–P20 SNc DA neurons with exogenous NMDA application (Dunah et al., 1998; Jones and Gibb, 2005; Brothwell et al., 2008; Suarez et al., 2010; Huang and Gibb, 2014) since all NMDA sEPSCs were sensitive to Ro 25-6981, but not all were sensitive to DQP 1105.

After P30, our results suggest that SNc neurons no longer express GluN2D-containing NMDA receptors, since NMDA sEPSCs were no longer either bursty or sensitive to DQP 1105. Transient expression of the GluN2D subunit mRNA and a peak of expression around P7 are features described in midbrain regions (Monyer et al., 1994; Dunah et al., 1996; Wenzel et al., 1996; Laurie et al., 1997; Liu and Wong-Riley, 2010), suggesting that GluN2D subunit-containing NMDA receptors play a role in these brain regions mainly during development (von Engelhardt et al., 2015). Consistent with these results, we demonstrate the dynamic remodeling of NMDA receptor subunit composition in SNc DA neurons during postnatal development with GluN2D subunits no longer present in young adult mice.

The key role of NMDAR-mediated currents at P4–P10 is also revealed by the large APV-sensitive (but DQP 1105-insensitive) burst of spikes evoked by STN stimulation in immature but not in P30+ SNc DA neurons. The lack of effect of DQP 1105 might result from the absence of NR2D subunit-containing NMDAR in the postsynaptic membrane of STN–SNc synapses. It could also result from the low participation of these NMDAR subtypes to the depolarization underlying the STN-evoked burst of spikes compared to the current through NR2B subunit-containing NMDARs and voltage-dependent calcium channels. Interestingly, a similar NMDAR-dependent burst was described in the SNc of P14–P21 mice (Hage and Khaliq, 2015), suggesting that NMDA EPSCs mature after P21 and before P30. This large P4–P10 STN-evoked burst of spikes did not result from a different basal tonic firing rate before the stimulation which tunes synaptic gain in SNc DA neurons (Hage and Khaliq, 2015). During the burst, the progressive increase in the depolarization underlying the burst of spikes might result from backpropagation in the dendrites of the first spikes in the burst. These backpropagated spikes, by increasing calcium influx in dendrites, amplify the amplitude of NMDA EPSPs evoked by STN stimulation (Hage and Khaliq, 2015).

## Author Contributions

EP, LAGC, FM, and RC performed the experiments. EP, LAGC, FM, and CH analyzed the data. CH designed the study and wrote the paper.

## Conflict of Interest Statement

The authors declare that the research was conducted in the absence of any commercial or financial relationships that could be construed as a potential conflict of interest.

## Supplementary material

Figure S1 **Representative confocal image of the SNc (coronal section) from a TH-GFP mouse showing TH-positive neurons labeled with Alexa A555 (left), GFP positive neurons (right) and merged image (center)**. Arrows show GFP-positive/TH-negative SNc neurons. Scale bar μ50 m.
