# Supplementary figures and images for: Glutamatergic synaptic currents of nigral dopaminergic neurons follow a postnatal developmental sequence
Source: Front Cell Neurosci. 2015 May 29;9:210. doi: 10.3389/fncel.2015.00210 (PMC4448554; doi:10.3389/fncel.2015.00210)

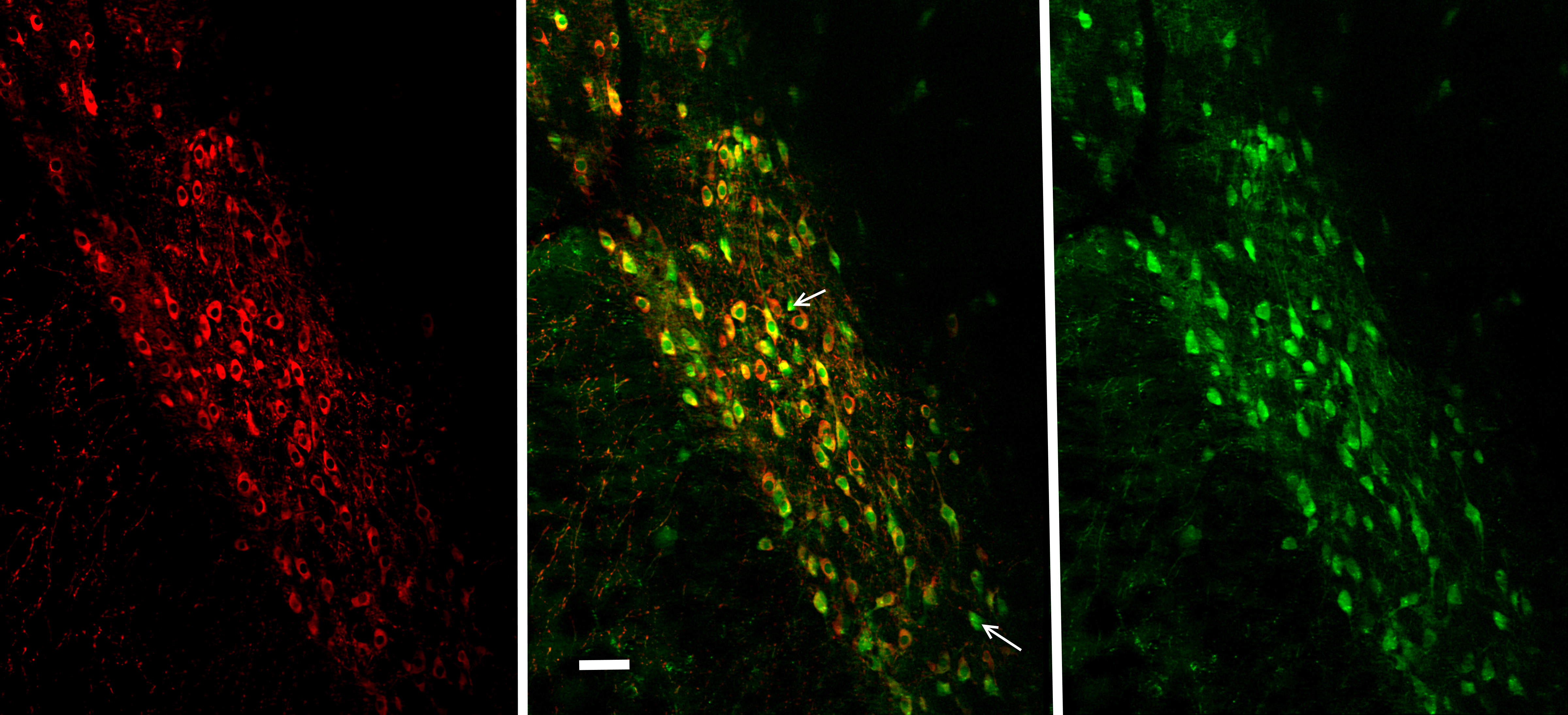

Supplement: Figure S1 — Representative confocal image of the SNc (coronal section) from a TH-GFP mouse showing TH-positive neurons labeled with Alexa A555 (left), GFP positive neurons (right) and merged image (center). Arrows show GFP-positive/TH-negative SNc neurons. Scale bar μ50 m. [file Image_1.TIF]
